# Left Bundle Branch Area Pacing versus Deep Septal Pacing in Patients After Transcatheter Aortic Valve Replacement

**DOI:** 10.1111/jce.70361

**Published:** 2026-05-03

**Authors:** Liangzhen Qu, Xueting Duan, Han Chen

**Affiliations:** ^1^ Department of Cardiology State Key Laboratory of Transvascular Implantation Devices, The Second Affiliated Hospital Zhejiang University School of Medicine Hangzhou Zhejiang China; ^2^ Department of Cardiology The Third Affiliated Hospital of Zhejiang Chinese Medical University Hangzhou Zhejiang China

**Keywords:** deep septal pacing, heart failure hospitalization, left bundle branch area pacing, NYHA class improvement, transcatheter aortic valve replacement

## Abstract

**Background:**

Left bundle branch area pacing (LBBAP) has been reported to improve long‐term clinical outcomes in patients requiring permanent pacemaker implantation (PPMI) after transcatheter aortic valve replacement (TAVR). Deep septal pacing (DSP) has emerged as a potential alternative to LBBAP.

**Objective:**

This study investigated whether short‐term and long‐term clinical outcomes differ between LBBAP and DSP in post‐TAVR patients.

**Methods:**

Consecutive patients undergoing LBBAP or DSP following TAVR were retrospectively included at our institution. Short‐term clinical outcomes (1‐year follow‐up) were assessed by echocardiographic measures of reverse remodeling and changes in QRS duration and N‐terminal pro–B‐type natriuretic peptide (NT‐proBNP) levels. For long‐term outcomes, the primary composite endpoint was all‐cause mortality or heart failure hospitalization (HFH). Secondary endpoints included HFH and improvement in New York Heart Association (NYHA) class (≥ 2 grades).

**Results:**

A total of 82 patients (39 LBBAP and 43 DSP) were observed for a mean duration of 731.8 days. There was no significant difference between two groups in the risk of primary endpoint (23.1% vs. 23.3%, adjusted hazard ratio [aHR] 0.61; 95% CI: 0.23–1.61, *p* = 0.315) and HFH (17.9% vs. 20.9%, aHR 0.64; 95% CI: 0.22–1.82; *p* = 0.402). However, LBBAP was a robust predictor of NYHA class improvement compared to DSP (53.8% vs. 27.9%, aHR 2.23; 95% CI: 1.03–4.87, *p* = 0.043), especially when left bundle branch (LBB) capture was independently confirmed (aHR 2.74, *p* = 0.006). Both modalities were similarly effective in improving electromechanical and biochemical parameters, including LVEF, LVEDD, QRS duration, and NT‐proBNP (all *p* > 0.05).

**Conclusion:**

LBBAP and DSP yield comparable risks for the primary composite endpoint and HFH, yet LBBAP provides superior symptomatic relief. Confirmation of left bundle branch capture is advisable to optimize clinical benefits.

Liangzhen Qu and Xueting Duan contributed equally to this manuscript.

## 1 Introduction

Transcatheter aortic valve replacement (TAVR) is a well‐established therapy for severe aortic stenosis (AS) at all levels of surgical risk [1, 2, 3, 4], and is also emerging for high‐risk patients with aortic regurgitation (AR) [5, 6]. Despite advancements in procedural skills and valve design, new‐onset conduction disturbances after TAVR remain common [7]. High‐grade atrioventricular block (AVB) and left bundle branch block (LBBB) often occur because the conduction system, which runs through the ventricular septum, is vulnerable to damage during the procedure [8]. It is reported that the incidence of periprocedural permanent pacemaker implantation (PPMI) ranges from 14.7% to 24% [9].

Currently, there are few studies on the optimal pacing strategy for patients with PPMI indications after TAVR. Right ventricular pacing (RVP) causes dyssynchrony and impaired left ventricular ejection fraction (LVEF), resulting in pacing‐induced cardiomyopathy (PICM) [10]. Previous studies showed that LBBB and wider QRS duration are independent predictors for PICM [11]. Pre‐existing LBBB was independently associated with increased risk of cardiovascular mortality after TAVR [12]. Since wide QRS at baseline or post‐TAVR is not rare, TAVR patients should be considered a susceptible population for PPMI [13]. In this scenario, left bundle branch area pacing (LBBAP) appears to be a superior pacing strategy due to its excellent ventricular resynchronization effect [14]. LBBAP is defined as the capture of the subendocardial region on the left side of the interventricular septum, with or without simultaneous conduction system capture [15], and includes left bundle branch pacing (LBBP) and left ventricular septal pacing (LVSP). During LVSP, despite the lack of direct capture, the left‐sided conduction system may be retrogradely engaged. Deep septal pacing (DSP) involves pacing deep in the septum without reaching the left ventricular (LV) subendocardium or directly capturing the left conduction system, yet it activates this system more rapidly than right ventricular septal pacing (RVSP) [16]. Due to the technical challenges and risk of procedural failure in scarred ventricle [17], some scholars have proposed DSP as a potentially feasible alternative [18]. The different lead depths among these three pacing strategies result in varying degrees of conduction system engagement. Accumulating evidence highlights the safety and feasibility of LBBAP in patients undergoing TAVR [19, 20, 21]. Recent studies suggest that direct LBB capture further reduces mortality compared to LVSP [22, 23], likely due to the more physiological LV activation sequence provided by LBBP. However, whether the physiological pacing gradient is associated with distinct clinical outcomes in post‐TAVR patients with severe ventricular hypertrophy and myocardial fibrosis remains unclear.

This study aimed to compare short‐term cardiac function improvement and long‐term clinical endpoints between LBBAP and DSP, and to further evaluate whether LBBP provides incremental benefits in this cohort.

## 2 Methods

### 2.1 Study Population

This was a single‐center, retrospective, observational study. It included all consecutive patients who underwent TAVR, had indications for PPMI, and attempted LBBAP within 30 days after the procedure at the Second Affiliated Hospital of Zhejiang University School of Medicine from April 2020 to October 2023. After numerous attempts to place the ventricular lead in the subendocardial area, DSP was considered as an alternative. Exclusion criteria were pre‐procedure permanent pacemaker, valve‐in‐valve procedures, procedural failure or death, concomitant infiltrative cardiomyopathy, and undergoing RVP or biventricular pacing (BiVP). The Institutional Review Board granted ethical approval for this work. The research reported in this article adhered to Helsinki Declaration guidelines.

### 2.2 PPMI after TAVR

**Figure 1:**
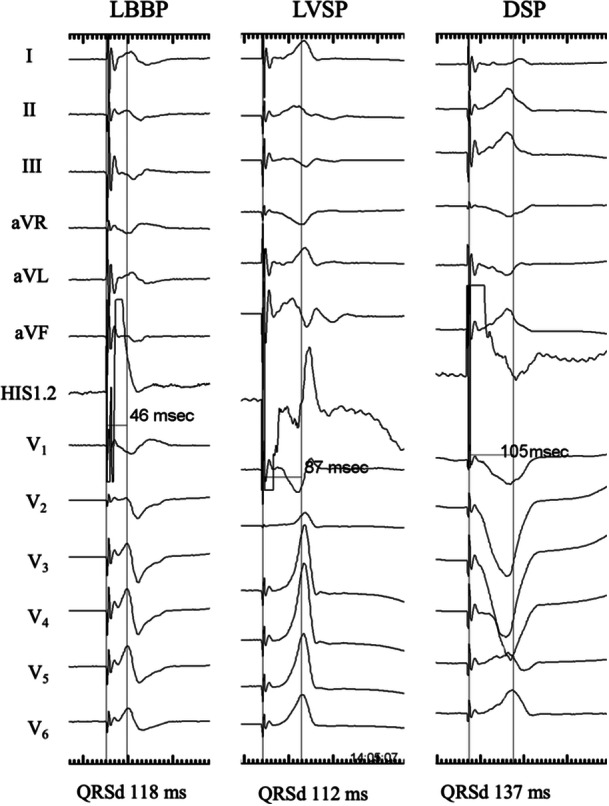
Examples of electrocardiograms by unipolar tip pacing in left bundle branch pacing (LBBP) with V_6_ R wave peak time (V6RWPT) of 46 ms and QRS duration (QRSd) of 118 ms, in left ventricular septal pacing (LVSP) with V6RWPT of 87 ms and QRSd of 112 ms, and in deep septal pacing (DSP) with V6RWPT of 105 ms and QRSd of 137 ms.

If a patient experienced high‐degree AVB, severe symptomatic bradycardia, hemodynamic instability or other circumstances where the electrophysiology team deemed a PPMI necessary, a pacemaker would be implanted following TAVR. LBBAP was attempted with the SelectSecure pacing lead (Model 3830, 69 cm; Medtronic, Minneapolis, MN) and a fixed‐curve sheath (C315 HIS; Medtronic) as previously described [24, 25]. In brief, the lead delivered through the sheath was screwed into the muscular intraventricular septum from the right to the left side. While advancing the lead, the paced QRS morphology was monitored using a multichannel recording system. LBBAP includes LBBP and LVSP. LBBP was defined by a right bundle branch block (RBBB) morphology of paced QRS in lead V_1_, along with at least 1 of the following criteria of left bundle branch (LBB) capture [26, 27, 28]: (1) V6 R‐wave peak time (V6RWPT) at high and low output of < 85 ms in patients with heart failure (HF) and < 75 ms in those with normal cardiac function or isolated RBBB; (2) the V6RWPT of < 80 ms with LBBB or IVCD; (3) the V_6_‐V_1_ interpeak interval of > 44 ms. LVSP was characterized by terminal R‐wave in V1, and absence of criteria for conduction system capture. DSP was defined as the absence of terminal r/R‐wave (QS or rS morphology), and no features of left conduction system capture [18, 29]. Lead position was confirmed by electrocardiogram (ECG) review of QRS morphology (Figure 1), with all ECGs reviewed by two independent members of the study team.

### 2.3 Data Collection and Follow‐Up

Baseline demographic information, clinical data, ECG, echocardiographic results, and TAVR procedure details were collected from the TAVR database at the Second Affiliated Hospital of Zhejiang University School of Medicine. The procedure duration of PPMI was recorded and the pacing parameters, such as pacing threshold, R‐wave amplitude and impedance were measured during the procedure. Paced QRS duration (QRSd) and V6RWPT were measured after the procedure. Device interrogation at the device clinic was conducted at 1 month, 3 months, and then at regular intervals of 6 months. Clinical follow‐up was carried out at 1 month, 12 months and annually afterwards. All patients were tracked for a minimum of 1 year. Long‐term clinical outcomes include all‐cause mortality, heart failure hospitalization (HFH) and improvement in New York Heart Association (NYHA) functional class. Short‐term clinical outcomes consisted of the changes in echocardiographic parameters, N‐terminal pro–B‐type natriuretic peptide (NT‐proBNP) and paced QRSd at 1 year after PPMI. LVEF was calculated from biplane Simpson's method. The echocardiographic response was defined as an increase in LVEF of ≥ 5%–20% and a reduction in left ventricular end‐diastolic diameter (LVEDD) of ≥ 10% [30]. All follow‐up echocardiograms were analyzed by experienced readers who were blinded to the patients' group assignment and designated lead position. NYHA class improvement occurred if it was decreased by ≥ 2 grades [31]. For patients with multiple episodes of HFH, only the first event was analyzed. Ventricular threshold, sensing, impedance, and pacing percentage were recorded from the last pacemaker interrogation at 1 year. PPMI procedure‐related complications include infection, lead failure, pocket hematoma, right ventricular perforation and so forth [32].

### 2.4 Statistical Analysis

Continuous variables were reported as mean ± SD or median (interquartile range). Means or medians were compared with a *t*‐test or Wilcoxon rank sum test. Categorical variables were presented as frequency or percentage and analyzed by *χ* ^*2*^ or Fisher's exact test. Within‐group comparisons were performed by means of 2‐tailed paired *t*‐test or Wilcoxon signed rank test. A Bonferroni correction was applied for multiple pairwise comparisons. A general linear model for repeated measures with interaction was used to compare the LVEF changes at different time points between two groups. Kaplan‐Meier (KM) survival curves with log‐rank tests were employed to compare the time to first event between LBBAP and DSP. The influence of two pacing strategies on clinical outcomes were estimated by Cox proportional hazards model. The variables considered clinically significant or that showed a univariate relationship with the outcome (*p*‐value < 0.1) were entered into multivariate analysis. A 2‐tailed *p*‐value < 0.05 was considered statistically significant. SPSS version 26.0 (IBM, Armonk, NY).

## 3 Results

### 3.1 Baseline Characteristics

A total of 82 patients were enrolled in this study, of which 30 (36.6%) patients underwent LBBP, 9 (11.0%) patients underwent LVSP, and 43 (52.4%) who underwent DSP. At baseline, the mean age of the entire cohort was 75 years, and 30 (36.6%) patients were female. The mean LVEF was 55.2% ± 10.4% and 8.5% (*n* = 7) had an LVEF of ≤ 40%. As shown in Table 1, the baseline features and comorbidities were similar among three groups. In the overall population, the mean Society of Thoracic Surgeons (STS) risk score was 4.3%. No significant differences were observed among the groups in the prevalence of pre‐TAVR arrhythmias (Atrial fibrillation [AF], 1st AVB, RBBB or LBBB) or the proportion of self‐expanding transcatheter heart valve (THV) implantation.

**Table 1:** Baseline characteristics.

	LBBP (*n* = 30)	LVSP (*n* = 9)	DSP (*n* = 43)	*p*
Age, y	73.8 ± 6.9	76.3 ± 5.1	75.6 ± 6.6	0.466
Sex, male	17 (56.7)	7 (77.8)	28 (65.1)	0.486
Body Mass Index, kg/m²	22.4 ± 3.4	21.2 ± 2.7	22.6 ± 3.2	0.511
NT‐proBNP, pg/mL	1708.0 (550.0–2345.0)	1066.5 (598.3–6213.8)	1914.5 (437.8–5438.5)	0.228
eGFR, mL/min/1.73 m²	52.2 ± 19.0	53.3 ± 13.1	57.5 ± 21.8	0.521
STS risk score, %	5.1 ± 5.0	3.9 ± 1.7	3.8 ± 2.4	0.283
Cardiac syncope	2 (6.7)	0 (0)	4 (9.3)	> 0.999
Medical history				
Hypertension	14 (46.7)	7 (77.8)	28 (65.1)	0.145
Diabetes mellitus	3 (10.0)	2 (22.2)	6 (14.0)	0.653
Stroke	2 (6.7)	0 (0)	2 (4.7)	> 0.999
Anemia	10 (33.3)	4 (44.4)	15 (34.9)	0.891
Coronary artery disease	11 (33.3)		24 (49.0)	0.160
PCI	2 (6.7)	1 (11.1)	4 (9.3)	0.868
NYHA class				0.474
I	1 (3.3)	0 (0)	1 (2.3)	
II	5 (16.7)	3 (33.3)	11 (25.6)	
III	16 (53.3)	5 (55.6)	27 (62.8)	
IV	8 (26.7)	1 (11.1)	4 (9.3)	
Pre‐TAVR Echocardiography				
Mean AV gradient, mmHg	35.0 (10.0–53.0)	18.5 (13.5–33.8)	40.5 (15.0–61.8)	0.217
AR ≥ moderate	21 (70.0)	7 (77.8)	30 (69.8)	0.886
AS ≥ moderate	16 (53.3)	5 (55.6)	29 (67.4)	0.449
TR ≥ moderate	5 (16.7)	1 (11.1)	5 (11.6)	0.893
LVEDD, mm	52.6 ± 7.8	56.8 ± 15.3	53.7 ± 11.0	0.628
LVEF, %	55.7 ± 10.4	59.2 ± 9.6	54.0 ± 10.5	0.365
LVEF < 50%	9 (30.0)	1 (11.1)	14 (32.6)	0.508
LVEF ≤ 40%	2 (6.7)	1 (11.1)	4 (9.3)	0.868
Pre‐TAVR ECG				
Atrial fibrillation	8 (26.7)	1 (11.1)	9 (20.9)	0.597
1st AVB	2 (6.7)	1 (11.1)	2 (4.7)	0.533
LBBB	0 (0)	0 (0)	2 (4.7)	0.612
RBBB	7 (23.3)	5 (55.6)	13 (30.2)	0.183
self‐expanding THVs	29 (96.7)	8 (88.9)	40 (93.0)	0.404

*Note:* Data are presented as mean ± SD or median (interquartile range) for continuous variables and numbers and percentages for categorical variables.

Abbreviations: AV = aortic valve, AVB = atrioventricular block, AR = aortic regurgitation, AS = aortic stenosis, DSP = deep septal pacing, ECG = electrocardiogram, eGFR = estimated glomerular filtration rate, LBBP = left bundle branch pacing, LBBB = left bundle branch block, LVEDD = left ventricular end‐diastolic diameter, LVEF = left ventricular ejection fraction, LVSP = left ventricular septal pacing, NT‐proBNP = N‐terminal pro–B‐type natriuretic peptide, NYHA = New York Heart Association, PCI = percutaneous coronary intervention, RBBB = right bundle branch block, STS = Society of Thoracic Surgeons, TAVR = transcatheter aortic valve replacement, THV = transcatheter heart valve.

*p value < 0.05 for comparison between LBBP, LVSP and DSP.

### 3.2 Pacing Characteristics and Parameters

As shown in Table 2, the most prevalent indication for PPMI was high‐degree or complete AVB (75.6%), and the majority of pacemakers implanted were dual chamber (91.5%). The QRSd prior to PPMI was comparable among LBBP, LVSP, and DSP (146.1 ± 25.3 ms in LBBP vs. 154.5 ± 11.1 ms in LVSP vs. 151.2 ± 16.3, *p* = 0.493). No significant differences in PPMI‐related complications and procedure time were observed in 3 groups. The LBBP group demonstrated significantly narrowest paced QRSd (116.9 ± 9.2 ms in LBBP vs. 123.0 ± 10.6 ms in LVSP vs. 128.2 ± 13.5 ms in DSP, *p* = 0.001) and the shortest V6RWPT (68.9 ± 12.6 ms in LBBP vs. 84.5 ± 11.4 ms in LVSP versus 79.6 ± 17.3 ms in DSP, *p* = 0.001). At implantation and follow‐up, pacing threshold, sensing, and impedance remained similar among the 3 groups. At the last follow‐up, 88.7% of the entire cohort exhibited ventricular pacing (VP) > 40%, and the median VP percentage was over 95% in 3 groups (96.7% in LBBP vs. 99.8% in LVSP versus 97.7% in DSP, *p* = 0.148).

**Table 2:** Pacing characteristics and parameters.

	LBBP (*n* = 30)	LVSP (*n* = 9)	DSP (*n* = 43)	*p*
Pre‐PPMI QRS, ms	146.1 ± 25.3	154.5 ± 11.1	151.2 ± 16.3	0.493
PPMI Indications				0.385
Complete/high‐degree AVB	25 (83.3)	8 (88.9)	29 (67.4)	
Severe symptomatic bradycardia	4 (13.3)	1 (11.1)	6 (14.0)	
HF + LBBB	1 (3.3)	0 (0)	2 (4.7)	
HF + QRS ≥ 150 ms	0 (0)	0 (0)	6 (14.0)	
**Procedural details**				
Device type				0.512
Single chamber	4 (13.3)	0 (0)	3 (7.0)	
Dual chamber	29 (86.7)	9 (100)	40 (93.0)	
Paced QRS duration, ms	116.9 ± 9.2	123.0 ± 10.6	128.2 ± 13.5a	0.001*
V6RWPT, ms	68.9 ± 12.6	84.5 ± 11.4a	79.6 ± 17.3a	0.001*
Ventricular pacing threshold, V at 0.4 ms	1.0 (0.6–1.0)	1.0 (0.9–1.0)	0.8 (0.6–1.0)	0.248
Ventricular impedance, Ω	965.9 ± 175.2	867.3 ± 113.6	921.2 ± 153.4	0.543
Ventricular sensing threshold, mV	10.9 (8.5–14.9)	10.6 (8.9–20.0)	14.3 (9.2–19.9)	0.172
Atrial pacing threshold, V	1.0 (0.9–1.5)	1.0 (1.0–1.3)	1.2 (1.0–1.5)	0.518
Atrial impedance, Ω	647.0 (515.0–856.5)	620.0 (520.0–777.0)	680.0 (565.3–779.3)	0.546
Atrial sensing threshold, mV	2.3 (1.4–4.4)	2.0 (1.8–3.6)	2.4 (2.0–3.7)	0.533
Procedure time, h	1.5 (1.2–1.8)	1.4 (1.0–1.7)	1.6 (1.3–1.8)	0.885
Complications	1 (3.0)	0 (0)	0 (0)	0.629
**1‐Year Follow‐up**				
Ventricular pacing threshold, V at 0.4 ms	0.8 (0.5–0.8)	0.8 (0.6–1.3)	0.8 (0.5–0.8)	0.243
Ventricular impedance, Ω	589.0 (532.0–646.0)	556.0 (485.3–629.5)	589.0 (551.0–666.0)	0.514
Ventricular sensing threshold, mV	20.0 (16.5–20.0)	11.0 (5.0–19.1)	14.8 (9.4–20.0)	0.061
Ventricular pacing, %	96.7 (62.8–99.8)	99.8 (99.4–100.0)	97.7 (82.5–99.9)	0.148

*Note:* Data are presented as mean ± SD or median (interquartile range) for continuous variables and numbers and percentages for categorical variables.

Abbreviations: AVB = atrioventricular block; DSP = deep septal pacing; HF = heart failure; LBBAP = left bundle branch area pacing; LBBB = left bundle branch block; LVAT = left ventricular activation time; PPMI = permanent pacemaker implantation.

a Indicates a statistically significant difference compared with the LBBP group. For paced QRS duration, DSP versus LBBP (*p* < 0.001); for V6RWPT, DSP versus LBBP (*p* = 0.005) and LVSP versus LBBP (*p* = 0.009).

* *p* value < 0.05 for the overall comparison among the three groups.

### 3.3 Short‐Term Clinical Outcomes

**Figure 2:**
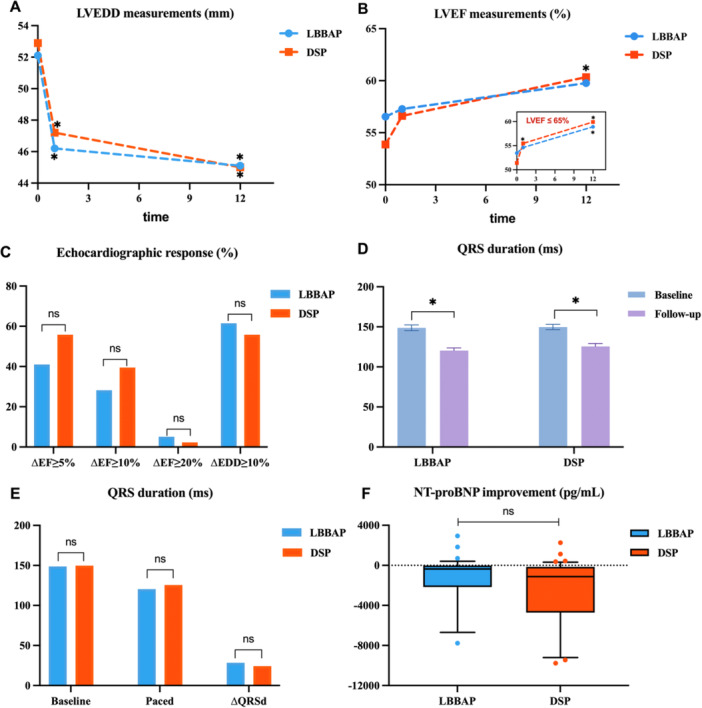
Short‐term (1‐year) clinical outcomes of left bundle branch area pacing (LBBAP) and deep septal pacing (DSP). (A) Trends in left ventricular end‐diastolic diameter (LVEDD) measurements during follow‐up. (B) Trends in left ventricular ejection fraction (LVEF) measurements during follow‐up. (C) Comparison of echocardiographic response (defined as a change in ejection fraction [∆EF] ≥ 5% or ≥ 10% or ≥ 20% and ∆EDD ≥ 10%). (D) Comparison of QRS duration at baseline and follow‐up in each group. (E) Comparison of baseline paced QRS duration, and changes in QRS duration (∆QRSd). (F) Comparison of changes in N‐terminal pro–B‐type natriuretic peptide (NT‐proBNP) levels. ns = non–statistically significant. *p‐value < 0.05 for comparison between baseline and follow‐up.

Echocardiographic evaluation demonstrated significant improvements in cardiac structure and systolic function, evidenced by a reduced end‐diastolic diameter and enhanced ejection fraction across both pacing modalities. At the 1‐year follow‐up, LVEDD significantly decreased from baseline in both the LBBAP group (from 52.3 ± 9.4 mm to 46.5 ± 9.0 mm, *p* < 0.001) and the DSP group (from 53.7 ± 11.0 mm to 46.5 ± 8.6 mm, *p* < 0.001). This improvement was primarily achieved within the first month, with a significant reduction observed at that timepoint in both groups (LBBAP: 47.7 ± 7.6 mm, *p* < 0.001; DSP: 47.8 ± 9.0 mm, *p* < 0.001 vs. baseline). No further significant change occurred from 1 month to 1 year in either group (LBBAP: *p* = 0.531; DSP: *p* = 0.416). In the DSP group, LVEF increased significantly from 53.9% ± 10.5% to 60.3% ± 9.1% (*p* < 0.001), while no significant increase was observed in the LBBAP group (from 56.5% ± 10.3% to 59.8% ± 9.5%, *p* = 0.084). Given the relatively high baseline LVEF in the LBBAP group (56.5% ± 10.3%), a subgroup analysis of patients with LVEF ≤ 65% showed that LVEF also increased from 53.2% ± 9.1% to 58.9% ± 9.9% (*p* = 0.004). Notably, LVEF in the LBBAP group remained stable during the first month and primarily improved from 1‐month and 1‐year follow‐ups (*p* = 0.027). Conversely, starting from a lower baseline (51.4% ± 9.1%), the DSP group demonstrated a steady upward trend, with improvements observed at both 1 month (*p* = 0.009) and 1 year (*p* < 0.001). As shown in Figures 2A and 2B, the recovery in LVEF and LVEDD over time did not differ significantly between 2 groups (LVEF: *p* = 0.282; LVEF ≤ 65%: *p* = 0.333; LVEDD: *p* = 0.843). The echocardiographic response (∆EF ≥ 5%–20%, ∆LVEDD ≥ 10%) at 1‐year follow‐up was also comparable in both groups (Figure 2C, Table S1).

At follow‐up, The QRSd significantly decreased from baseline in both the LBBAP group (from 148.2 ± 22.6 to 120.5 ± 21.1 ms, *p* < 0.001) and the DSP group (from 150.2 ± 21.8 to 125.3 ± 24.4 ms, *p* < 0.001) (Figure 2D), while no marked difference was observed in the extent of QRS shortening (∆QRSd) between 2 groups (*p* = 0.516) (Figure 2E, Table S1). Similarly, both groups demonstrated a reduction in NT‐proBNP (LBBAP: from 1050.5 [288.0–2332.3] to 360.3 [180.3–707.5] pg/mL, *p* < 0.001; DSP: from 2013.0 [733.5–5364.0] to 400.0 [173.0–838.7] pg/mL, *p* < 0.001), with no significant difference in the degree of reduction between groups (*p* = 0.097) (Figure 2F, Table S1). Given the comparable recovery between LBBAP and DSP, we further explored whether the direct LBB capture impacted outcomes. Because the LVSP subgroup was too small for independent analysis (*n* = 9) and shares the electrophysiological nature of DSP, these patients were pooled to form the non‐LBBP group. The regrouping analysis (LBBP vs. LVSP + DSP) revealed no significant differences between the two groups regarding electrophysiological or biomarker improvements (all *p* > 0.05, Table S2).

### 3.4 Long‐Term Clinical Outcomes

Table 3 presents the incidence of primary and secondary endpoints of the entire population in the two groups. After a mean follow‐up duration of 731.8 ± 339.7 days, the incidence of the primary composite endpoint was 23.2%, with HFH of 19.5%. Furthermore, over a median follow‐up of 388.5 (353.3–738.0) days, the rate of NYHA class improvement (∆NYHA ≥ 2 grades) was 40.2%.

**Table 3:** Long‐term clinical outcomes.

Outcomes	Model 1: Anatomical region		Model 2: LBB capture			
	LBBAP (*n* = 39)	DSP (*n* = 43)	*p*	LBBP (*n* = 30)	Non‐LBBP (*n* = 52)	*p*
**Follow‐up duration, days**	728.4 ± 325.8	734.8 ± 355.8	0.932	703.6 ± 321.3	748 ± 352.0	0.572
All‐cause mortality or HFH	9 (23.1)	10 (23.3)	0.985	7 (23.3)	12 (23.1)	0.979
HFH	7 (17.9)	9 (20.9)	0.734	5 (16.7)	11 (21.2)	0.621
**Follow‐up duration, days** ‡	374.0 (348.0–721.0)	625.0 (357.0–750.0)	0.341	365.5 (346.5–722.3)	599.0 (357.3–749.3)	0.211
∆NYHA ≥ 2 grades†	21 (53.8)	12 (27.9)	0.017*	18 (60.0)	15 (28.8)	0.006*

*Note:* Data are presented as median (interquartile range) or mean ± SD for continuous variables, and numbers (percentages) for categorical variables.

Abbreviations: DSP = deep septal pacing, HFH = heart failure hospitalization, LBBAP = left bundle branch area pacing, LBBP = left bundle branch pacing, NYHA = New York Heart Association.

* *p* value < 0.05 for comparison between the two groups within the respective model.

^‡^ Indicates the specific follow‐up duration for the assessment of NYHA class improvement.

^†^ ∆NYHA ≥ 2 grades: ≥ 2 grades improvement in NYHA class from baseline.

**Figure 3:**
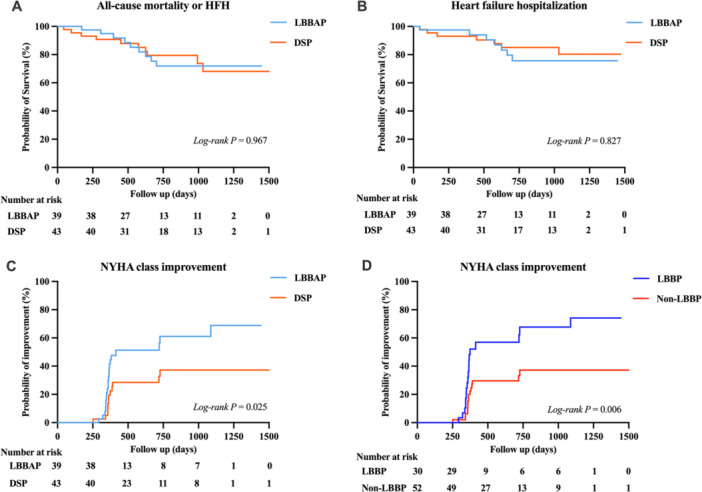
Kaplan‐Meier curves for the unadjusted cumulative probabilities of primary and secondary clinical endpoints among different pacing strategies. Kaplan‐Meier curves for the unadjusted survival free from (A) all‐cause mortality or heart failure hospitalization (HFH) and (B) HFH alone between left bundle branch area pacing (LBBAP) and deep septal pacing (DSP). Cumulative probabilities of NYHA class improvement comparing (C) LBBAP versus DSP, and (D) LBBP versus non‐LBBP (including left ventricular septal pacing and deep septal pacing).

No significant differences were observed in the incidences of the primary composite endpoints (23.1% vs. 23.3%, *p* = 0.985) and HFH (17.9% vs. 20.9%, *p* = 0.734) between LBBAP and DSP (Table 3). KM survival curves also indicated similar risks of primary endpoint (*Log‐rank p* = 0.967) and HFH (*Log‐rank p* = 0.827). Conversely, the incidence of NYHA class improvement was significantly higher in the LBBAP group compared to the DSP group (53.8% vs. 27.9%, *p* = 0.017). Based on KM survival curves, LBBAP achieved clinical improvement in NYHA class (≥ 2 grades) at a significantly higher rate than those receiving DSP (*Log‐rank p* = 0.025) (Figure 3).

Multivariate Cox regression models (Table 4) were further applied to explore long‐term effect. After adjustment for sex, age, STS score and all other variables with a *p*‐value < 0.1 in univariate analysis, the adjusted risk of the primary outcome and HFH remained comparable between LBBAP and DSP (adjusted hazard ratio [aHR] 0.61, 95% CI: 0.23–1.61, *p* = 0.315; aHR 0.64, 95% CI: 0.22–1.82, *p* = 0.402, respectively). Regarding the improvement in NYHA class, LBBAP was still independently associated with a higher probability of improvement compared to DSP (aHR 2.23; 95% CI: 1.03–4.87, *p* = 0.043), indicating that the likelihood of symptomatic relief was approximately twice as high in the LBBAP group.

**Table 4:** Influence of two pacing strategies on the long‐term clinical outcomes.

	Univariate analysis		Multivariate analysis	
	HR (95% CI)	*p*	HR(95% CI)	*p*
**Primary outcome: All‐cause mortality or HFH**				
LBBAP versus DSP	1.02 (0.41–2.51)	0.967	0.61 (0.23–1.61)	0.315
LBBP versus non‐LBBP	1.14 (0.47–2.76)	0.767	0.71 (0.27–1.91)	0.500
**Secondary outcome 1: HFH**				
LBBAP versus DSP	0.90 (0.33–2.41)	0.827	0.64 (0.22–1.82)	0.402
LBBP versus non‐LBBP	0.95 (0.36–2.52)	0.911	0.56 (0.19–1.68)	0.298
**Secondary outcome 2: ∆NYHA** ≥ **2 grades** †				
LBBAP versus DSP	2.21 (1.09–4.51)	0.029*	2.23 (1.03–4.87)	0.043*
LBBP versus non‐LBBP	2.55 (1.28–5.07)	0.008*	2.74 (1.33–5.65)	0.006*

*Note:* In multivariate analysis, we adjusted age, sex, STS score, and all other variables with a *p*‐value of < 0.1 in univariate analysis (hypertension, diabetes mellitus, coronary artery disease, valvular heart disease, post‐TAVR LBBB, eGFR, LVEDD, LVEF, NT‐proBNP, pre‐RBBB, atrial fibrillation).

Abbreviation: CI = confidence interval, DSP = deep septal pacing, HFH = heart failure hospitalization, HR = hazard ratio, LBBAP = left bundle branch area pacing, LBBP = left bundle branch pacing, NYHA = New York Heart Association.

* *p*‐value < 0.05 for comparison between the two groups.

^†^ ∆NYHA ≥ 2 grades: ≥ 2 grades improvement in NYHA class from baseline.

We applied the aforementioned physiological re‐grouping (LBBP vs. non‐LBBP) to the long‐term endpoints. Consistent with the short‐term findings, the primary composite endpoint and HFH remained comparable between the two groups (all *p* > 0.05, Table 4). Notably, this re‐grouping analysis yielded a higher adjusted hazard ratio (aHR 2.74; 95% CI: 1.33–5.65, *p* = 0.006) compared to the initial anatomical grouping (aHR 2.74 vs. 2.23), indicating a stronger association between direct LBB capture and symptomatic improvement.

## 4 Discussion

This real‐world, retrospective study compared the clinical outcomes of LBBAP and DSP in patients undergoing TAVR. Our main findings were as follows: (1) There was no significant difference in the incidence of HFH and the composite endpoint of all‐cause death and HFH between LBBAP and DSP. (2) LBBAP was associated with a higher rate of NYHA class improvement (≥ 2 grades) compared to DSP, with direct LBB capture serving as a robust independent predictor of this long‐term symptomatic recovery. (3) Both LBBAP and DSP yielded significant and comparable short‐term echocardiographic reverse remodeling (∆EF ≥ 5%–20%, ∆LVEDD ≥ 10%), NT‐proBNP reduction, and QRS shortening.

Previous studies have reported that the development of LBBB or AV block after TAVR is associated with diminished improvement in LV function and increased mortality [33, 34]. Due to extensive injury to the conduction system by self‐expanding THV implantation, LBBAP has more advantages than HBP in patients after TAVR [20, 21, 33, 34]. Recently, a multicenter retrospective study confirmed that LBBAP is superior to RVP in terms of reduced heart failure hospitalization (HFH) and better LVEF [35]. An ongoing randomized controlled trial (RCT) is designed to further confirm the benefits of LBBP over RVP after TAVR (RAFT‐TAVR PACE, NCT 06857201). Several studies have shown that LBBAP results in more physiological LV activation in patients with normal His‐Purkinje conduction and is associated with preserved or improved LV function in those with LBBB [36, 37]. Therefore, LBBAP appears to be the first‐line option for patients who need PPMI after TAVR, particularly those with a high ventricular pacing burden (> 20%) [27, 38]. However, long‐term LV pressure overload causes diffuse myocardial fibrosis and lesions of the conduction system [39, 40], elevating the technical complexity of LBBP in TAVR patients. This may partially explain the relatively high proportion of DSP (52.4%) observed in our cohort. Unlike patients with spontaneous high‐degree AV block, those undergoing TAVR have often developed electromechanical remodeling prior to the onset of iatrogenic conduction disturbances. Cardiac magnetic resonance imaging demonstrates that aortic stenosis drives early, diffuse interstitial fibrosis and collagen deposition, which ultimately results in irreversible mid‐wall scarring in approximately 12% of patients [41, 42]. Previous studies have demonstrated that patients requiring PPMI following TAVR frequently exhibit various degrees of left ventricular outflow tract (LVOT) calcification [43], with case reports indicating that this calcific burden can even extend into the septal region [44, 45]. The inherently floppy 3830 lumenless lead is susceptible to “endocardial barrier effect or entanglement” [44, 46], causing an inability to penetrate the deep septum, which accounts for up to 41.8% of procedural failures [47]. Furthermore, collagen deposition disrupts intercellular electrical coupling and induces conduction anisotropy [48]. Consequently, the prolonged latency interval is ‘filled’ with local myocardial depolarization, which partially conceals LBB capture and manifests as ‘functional’ DSP or LVSP. Additionally, the ~30% prevalence of baseline RBBB—an established predictor of LBBP failure [49] —coupled with varying operator proficiency, further contributed to the observed DSP rate. Due to the absence of direct conduction system capture, the ventricular resynchronization of DSP and LVSP may be worse than LBBP. This is consistent with our results, which demonstrated that DSP and LVSP had a longer paced QRS and V6RWPT compared to LBBP. Currently, DSP has not been widely studied. A previous multicenter retrospective study demonstrated that DSP failed to significantly improve cardiac function (LVEF and LVEDD) and was linked to a higher risk of all‐cause death and HFH [29]. However, this study was limited by a CRT‐indicated population and a small DSP sample size (*n* = 14).

To our knowledge, this is the first study to specifically compare the clinical outcomes of LBBAP and DSP. Our study demonstrated comparable risks of the primary composite endpoint and HFH, along with similar improvements in cardiac structure and systolic function between LBBAP and DSP. This may be primarily explained by TAVR‐induced LV reverse remodeling, evidenced by reduced LV mass and improved LVEF [50], which may mask the additional benefits of different pacing modalities [8, 35]. Secondly, the 30‐day PPMI was actually a protective factor and did not show any increase in death (overall or cardiovascular) or HFH [51], LBBAP or DSP may not remarkably impact the survival rate in this context. Thirdly, the limited sample size and moderate follow‐up (~2 years), coupled with the lack of routine programmed stimulation to exclude functional DSP, may have diluted inter‐group differences. Finally, unlike previous studies focused on the population with heart failure with reduced ejection fraction (HFrEF) [22, 52, 53], our study population had predominantly preserved systolic function (with a mean LVEF of 55.2% ± 10.4%; only 8.5% were HFrEF). In this case, the differential effects of LBBAP versus DSP may be attenuated. Existing literature indicates that for patients with preserved ejection fraction, LBBP and LVSP were comparable in mechanical synchrony and HFH rate [54]. Although the small LVSP sample size (*n* = 9) prevented a direct comparison, our results are in line with these findings, suggesting that the benefit of LBB capture may be less pronounced in patients with preserved systolic function. The high baseline LVEF also makes it difficult to show a significant recovery. Under these circumstances, NYHA class reduction, despite its inherent subjectivity, remains a powerful clinical predictor of future adverse events [55], and may better reflect patients' quality of life and clinical efficacy. Our study demonstrated that LBBAP was associated with twice the rate of NYHA class improvement (≥ 2 grades) compared to DSP (aHR 2.23; 95% CI: 1.03–4.87, *p* = 0.043). This may be attributed to the superior electromechanical synchrony resulting from left conduction system capture, given that even LVSP may retrogradely engage the left conduction system. Indeed, when LVSP cases were excluded, LBBP further magnified this symptomatic relief (aHR 2.74; 95% CI: 1.33–5.65, *p* = 0.006). These findings align with the long‐term benefits of physiological capture seen in large registries like *MELOS RELOADED* [23], despite potential dilution by functional DSP or LVSP. The female representation was limited (38.5% in LBBAP vs. 34.9% in DSP), reflecting the inherently higher incidence of post‐TAVR PPMI among males [56, 57]. Currently, it remains controversial whether sex influences the prognostic benefit of LBBAP [58, 59], possibly resulting from differences in study populations. In our study, we observed no significant association between sex and the composite endpoint in either group (LBBAP *p* = 0.936, DSP *p* = 0.920). Sex was also adjusted as a covariate in multivariate regression model to mitigate its potential confounding. Furthermore, the retrospective design inherently introduces missing data. We addressed minor baseline missingness (< 5%) via mean imputation and follow‐up NT‐proBNP missingness (< 15%, primarily due to clinical BNP testing preferences) via multiple imputation, yielding results consistent with complete‐case analyses. Additionally, this real‐world study was not randomized but driven by operator's judgement. The homogeneity between the study groups could not be ensured entirely although confounding factors were adjusted by several statistical methods.

In this study, we demonstrated that LBBAP provided superior long‐term symptomatic relief compared to DSP in post‐TAVR patients, primarily driven by LBB capture. In contrast, the risks of primary composite endpoint and HFH were comparable in both groups. While direct LBB capture is optimal for maximizing long‐term outcomes, it remains technically challenging in TAVR patients with established remodeling, making LBBAP a practical procedural endpoint. Alternatively, DSP offers a shorter learning curve alongside reduced procedural and fluoroscopy times. These efficiencies may be advantageous for some vulnerable populations, such as the elderly or those with renal dysfunction. Given the typically preserved cardiac function in patients at the compensated stage of aortic valve disease, DSP could serve as a reasonable fallback. If validated by future large‐scale trials, this tiered approach will optimize pacing strategies and broaden the adoption of CSP.

## 5 Conclusion

LBBAP provides superior symptomatic relief compared to DSP in post‐TAVR patients, despite comparable long‐term clinical outcomes. This strengthens the use of LBBAP in this population while awaiting ongoing randomized trials. Confirmation of true LBB capture is recommended for optimal benefit. Both modalities similarly improve cardiac structure, QRS duration, and NT‐proBNP levels.

## Author Contributions

Liangzhen Qu was responsible for the study design, statistical analysis and drafted the primary manuscript, Xueting Duan was in charge of critical revision and completing the figure illustrations, Han Chen raised the initial conception of the study and polished the manuscript. All authors reviewed and approved the final version of the manuscript.

## Conflicts of Interest

The authors declare no conflicts of interest.

## Supporting information


**Supporting File:** jce70361‐sup‐0001‐supplement_materials.docx.

## Data Availability

The data that support the findings of this paper are available from the corresponding author (Han Chen) upon reasonable request.
