# Supplementary material for: Left Bundle Branch Area Pacing versus Deep Septal Pacing in Patients After Transcatheter Aortic Valve Replacement
Source: J Cardiovasc Electrophysiol. 2026 May 3;37(7):1421–31. doi: 10.1111/jce.70361 (PMC13372404; doi:10.1111/jce.70361)
Supplement: Supplementary file 1 — Supporting File: jce70361‐sup‐0001‐supplement_materials.docx. [file JCE-37-1421-s001.docx]

**Table S1.** Echocardiographic, electrocardiographic, and biomarker responses based on anatomical region (Model 1)

| **Variables** | **LBBAP (n = 39)** | **DSP (n = 43)** | ***P* value** |
| --- | --- | --- | --- |
| **Echocardiographic response** |  |  |  |
| ∆LVEF ≥ 5% | 16 (41.0) | 24 (55.8) | .181 |
| ∆LVEF ≥ 10% | 11 (28.2) | 17 (39.5) | .280 |
| ∆LVEF ≥ 20% | 2 (5.1) | 1 (2.3) | .602 |
| ∆LVEDD ≥ 10% | 24 (61.5) | 24 (55.8) | .599 |
| **Electrocardiographic and biomarker changes** |  |  |  |
| ∆QRS duration, ms | 28.3 ± 27.9 | 24.2 ± 28.9 | .516 |
| ∆NT-proBNP, pg/mL | -358.0 (-2087.0 to -12.5) | -1118.0 (-4533.4 to -178.0) | .097 |

**Table S2.** Echocardiographic, electrocardiographic, and biomarker responses based on left bundle branch capture (Model 2)

| **Variables** | **LBBP (n = 30)** | **Non-LBBP (n = 52)** | ***P* value** |
| --- | --- | --- | --- |
| **Echocardiographic response** |  |  |  |
| ∆LVEF ≥ 5% | 13 (43.3) | 27 (51.9) | .454 |
| ∆LVEF ≥ 10% | 10 (33.3) | 18 (34.6) | .906 |
| ∆LVEF ≥ 20% | 2 (6.7) | 1 (1.9) | .551 |
| ∆LVEDD ≥ 10% | 18 (60.0) | 30 (57.7) | .838 |
| **Electrocardiographic and biomarker changes** |  |  |  |
| Baseline QRS duration, ms | 146.5 ± 24.6 | 150.9 ± 20.1 | .374 |
| Paced QRS duration at 1 year, ms | 118.4 ± 18.1 | 125.8 ± 24.8 | .155 |
| ∆QRS duration, ms | 28.1 ± 28.7 | 25.1 ± 28.3 | .650 |
| Baseline NT-proBNP, pg/mL | 1586.0 (430.5–2336.5) | 1653.5 (411.5–5289.5) | .153 |
| NT-proBNP at 1 year, pg/mL | 381.0 (192.2–729.0) | 390.0 (157.5–823.3) | .638 |
| ∆NT-proBNP, pg/mL | -437.0 (-2540.5 to -11.3) | -998.9 (-4347.4 to -51.3) | .366 |

Data are presented as mean ± SD or median (interquartile range) for continuous variables, and as numbers (percentages) for categorical variables. The symbol ∆ indicates the absolute change from baseline to the 1-year follow-up.

DSP = deep septal pacing; LBBAP = left bundle branch area pacing; LVEDD = left ventricular end-diastolic diameter; LVEF = left ventricular ejection fraction; NT-proBNP = N-terminal pro-B-type natriuretic peptide.
